# Consumption of Solnul^™^ Resistant Potato Starch Produces a Prebiotic Effect in a Randomized, Placebo-Controlled Clinical Trial

**DOI:** 10.3390/nu15071582

**Published:** 2023-03-24

**Authors:** Jason R. Bush, Joshua Baisley, Scott V. Harding, Michelle J. Alfa

**Affiliations:** 1 MSP Starch Products Inc., Carberry, MB R0K 0H0, Canada; 2 Nutrasource Pharmaceutical and Nutraceutical Services, Guelph, ON N1G 0B4, Canada; 3 Department of Biochemistry, Faculty of Science, Memorial University of Newfoundland, St. John’s, NL A1C 5S7, Canada; 4 AlfaMed Consulting, Winnipeg, MB R2M 5M3, Canada

**Keywords:** prebiotic, constipation, diarrhea, potato, resistant starch, *Bifidobacterium*, *Akkermansia*, microbiome

## Abstract

The effects of resistant starch at high doses have been well-characterized, but the potential prebiotic effects of resistant starch at doses comparable to oligosaccharide prebiotics have not been evaluated. A three-arm randomized, double-blind, placebo-controlled clinical trial was conducted to evaluate the effect of 3.5 g and 7 g daily doses of Solnul^™^ resistant potato starch (RPS) on beneficial populations of gut bacteria and stool consistency after a 4-week period. The relative abundance of *Bifidobacterium* and *Akkermansia* was determined by employing 16Sv4 sequencing of stool samples. To assess the effect of RPS on laxation and bowel movements, stools were recorded and scored using the Bristol Stool Form Scale. Participants consuming 3.5 g/day of RPS experienced significantly greater changes in Bifidobacterium and Akkermansia compared to the placebo after 4 weeks. The number of diarrhea- and constipation-associated bowel movements were both significantly lower in the 3.5 g RPS arm compared to the placebo group. Participants consuming 7 g of RPS responded similarly to those in the 3.5 g arm. Our analyses demonstrate that Solnul^™^ RPS has a prebiotic effect when consumed for 4 weeks at the 3.5 g per day dose, stimulating increases in beneficial health-associated bacteria and reducing diarrhea- and constipation-associated bowel movements when compared to the placebo group.

## 1. Introduction

Resistant starch (RS) is defined as ‘the sum of starch and starch-degradation products that, on average, reach the human large intestine’ [1]. Most RS is classified into five types based on the natural or manufactured form, with RS Type 1 (RS1) including starch physically inaccessible to amylase, RS Type 2 (RS2) including naturally occurring starch that resists amylase due to the shape of the granule, RS Type 3 (RS3) including heated-then-cooled starches that adopt a retrograded crystalline structure that resists amylase degradation, RS Type 4 (RS4) including chemically modified starches that resist amylase degradation, and RS Type 5 (RS5) including starch-lipid complexes that evade amylase degradation [2]. Uncooked potato, green banana, and high amylose maize are all naturally occurring sources of RS2, while cooked and cooled pasta and cooled baked potato are sources of RS3 [3]. RS2 and RS4 are recognized as forms of dietary fiber in Canada (Health Canada) and the United States (Food and Drug Administration), though the fermentability of RS4 and its effect on gut microbiota vary depending on the material it was derived from [4]. Resistant potato starch (RPS) is exceptional among the various types of dietary fiber in that it is insoluble but fully fermentable [5], the latter being a characteristic that is typically associated with soluble fibers.

While Australian dietary fiber levels approached those recommended by the National Health and Medical Research Council, bowel cancer rates remained high, and subsequent investigations revealed that RS levels in the Australian diet remained low (3–9 g per day; Commonwealth Scientific and Industrial Research Organization, Australia), suggesting that RS may play a valuable role among various forms of dietary fiber [6,7]. Americans consume approximately 4 g of RS per day, mostly RS2 and RS3 [8], with suggested dietary targets ranging from 15–20 g per day for optimal metabolic and bowel health [8,9]. Closing this gap through diet is challenging, given that most foods contain only small amounts of RS [3]. Furthermore, the food commonly consumed by Americans that contributes the most dietary RS is French fries [8], a food associated with negative health consequences, making it unreasonable to use such dietary sources to close the gap between current RS intake and suggested dietary targets.

Clinical trials evaluating the health benefits of RS fortification have typically investigated high doses in food formats, producing benefits including laxation [10,11,12,13], lower fasting blood glucose or HbA1c [14,15,16,17,18], and improved insulin metabolism [14,17,19,20,21,22,23,24,25,26]. Evidence from animal and human studies indicates that short-chain fatty acids derived from RS fermentation can attenuate inflammatory and oxidative stress pathways, which may mitigate the progression of diabetic kidney disease [27], an active area of investigation [28]. Other studies have investigated the effects of RS consumption on the composition of the gut microbiota, which is generally characterized by an increased abundance of *Bifidobacterium* for RPS and an increased abundance of *Ruminococcus* for high amylose maize starch [29]. Only a subset of RS2 clinical trials has measured both a health benefit and specific changes in the microbiome [14,30,31,32,33], which are required to demonstrate a prebiotic effect [34].

While current evidence suggests that higher doses of RS2 (i.e., 15–30 g/day) are needed to achieve metabolic improvements [8,14], we hypothesized that lower doses of RPS might provide a prebiotic effect by enhancing the abundance of beneficial bacteria while improving stool consistency. To test this, we designed and performed a study evaluating these effects in response to 3.5 g or 7 g RPS per day for four weeks in healthy individuals in a randomized, double-blind, placebo-controlled clinical trial.

## 2. Materials and Methods

### 2.1. Investigational Product

The resistant starch (RS) used in this study was Solnul^™^ (MSP Starch Products Inc., Carberry, MB, Canada), an unmodified resistant potato starch (RPS; a form of RS Type 2) produced via a unique processing method that preserves RS resulting in a stable RS content of 60% (AOAC 2002.02). The placebo used was fully digestible corn starch (Amioca; Ingredion, Brampton, ON, Canada) and contained no RS [30]. Investigational products were packaged in identical single-serving sachets and labeled in accordance with the randomization codes from Nutrasource Pharmaceutical and Nutraceutical Services (Guelph, ON, Canada) by personnel not involved in the collection of study data to ensure blinding of the study.

### 2.2. Study Design and Participant Selection

This study was conducted between 30 October 2019 and 6 January 2020 in Guelph, ON, Canada, and recruited participants from the general population in Guelph and the surrounding area. On-site monitoring was conducted according to the Clinical Monitoring Plan. The data management and statistical analyses for this study were conducted according to Standard Operating Procedures based on the International Council for Harmonization (ICH; www.ich.org, accessed on 26 October 2019), Health Canada Natural Health Product Regulations (https://www.canada.ca/en/health-canada/services/drugs-health-products/natural-non-prescription/legislation-guidelines/guidance-documents/clinical-trials.html, accessed on 26 October 2019), and the Food and Drug Administration (FDA) regulations and guidance documents (https://www.fda.gov/regulatory-information/search-fda-guidance-documents/clinical-trials-guidance-documents, accessed on 26 October 2019).

The study protocol and related documents were approved by Canadian Shield Ethics Review Board (REB Tracking Number: 19-10-001; Burlington, ON, Canada) on 29 October 2019. A sample size calculation determined that a minimum total sample of *n* = 20 in each arm would have power = 0.80 to detect a Cohen’s *f* effect size of 0.33 between treatment arms and placebo with a nominal α = 0.05 for similar gastrointestinal and microbiome-related outcomes [14,30]. To allow for participant attrition, a total sample size of *n* = 25 was selected. This study was conducted in accordance with the protocol and with the consensus ethical principles derived from international guidelines, including the Declaration of Helsinki and Council for International Organizations of Medical Sciences International Ethical Guidelines, applicable ICH Good Clinical Practice guidelines, and applicable local and federal laws and regulations. The trial is registered at ClinicalTrials.gov (NCT05242913).

The investigator or investigator’s representative explained the nature of the study to the participant or the participant’s legally authorized representative and answered all questions regarding the study. Participants were informed that their participation was voluntary and that any study report or publication of the study results would not disclose the participant’s identity without specific consent. Participants wishing to participate in the study, or their legally authorized representative, were required to sign a statement of informed consent that met the requirements of local regulations, ICH guidelines, and the research ethics board. The authorized person obtaining the informed consent also signed the informed consent form (ICF). A copy of the ICF was provided to the participant or the participant’s legally authorized representative. Written informed consent was obtained prior to any study-related procedures.

Participants enrolled in this study were healthy adults between 18–69 years of age with a body mass index (BMI) of 18.0 to 34.9 kg/m^2^ (Table 1). Eligible participants agreed to not any vitamins, minerals, or dietary supplements 14 days prior to the randomization visit until the completion of the final visit since consumption of these products could impact the results for the investigational product. Participants were counseled to follow their habitual diet throughout the study period. Individuals with a BMI over 34.9 kg/m^2^ were excluded as their health, and any related metabolic changes may impact the results of the study independent of the RPS or placebo. Additionally, individuals with a diagnosis of irritable bowel syndrome (IBS), dyspepsia, significant gastrointestinal disorders or other major diseases were excluded.

**Figure 1:**
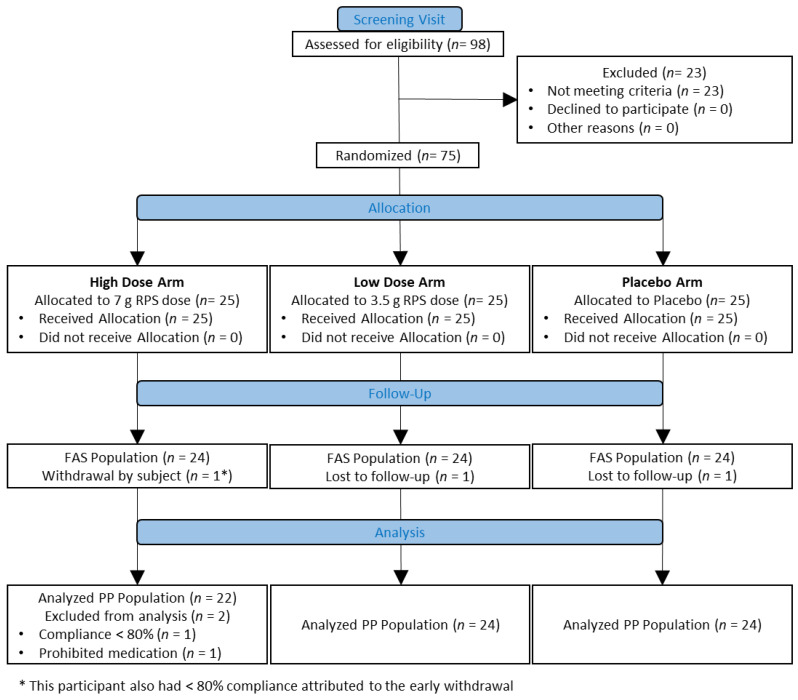
CONSORT flow diagram. Ninety-eight people were assessed for eligibility, of which 75 were randomized to one of three treatment arms: High dose (7 g Resistant potato starch (RPS) per day), low dose (3.5 g RPS per day), or placebo (Corn starch). Full analysis set (FAS) and per protocol (PP) populations are indicated, including reasons for participant attrition.

This was a randomized, double-blind, placebo-controlled three-arm parallel-group study (CONSORT Diagram; Figure 1). A total of 98 participants were screened for eligibility, of which 75 participants (25 participants per study arm) were eligible to be enrolled in the study. The study included a screening visit from 30 days up to 14 days prior to randomization, a run-in period of 14–17 days prior to randomization, a baseline visit (Day 0) during which the randomization was performed, and two subsequent study visits at Weeks 1 and 4, respectively (Table 2). During the screening visit, stool/bowel movement records and stool collection instructions and materials (including two fecal sample collection containers (DNA Genotek, Ottawa, ON, Canada)) were provided. During the run-in period, participants recorded their daily bowel habits for 14–17 days in a diary. As fecal sample collection was spontaneous, participants who produced a fecal sample that was collected prior to 72 h before the end of the study were documented, but this was not considered a protocol deviation.

Participants collected a fecal sample within 72 h prior to the Day 0 visit and transferred it to the clinic site within 24 h of collection. Stool samples for microbiome analysis were collected using the OMNIgene-Gut kits as per the manufacturer’s instructions. During the baseline visit (Day 0), the participants were randomized to receive one of three study interventions as indicated by the randomization scheme: High dose (7 g resistant potato starch (RPS)), low dose (3.5 g RPS combined with 3.5 g digestible corn starch), or placebo (7 g digestible corn starch). The randomization scheme was generated by Nutrasource Pharmaceutical and Nutraceutical Services using SAS 9.4 PROC PLAN with Seed Number: 1887363180. Seventy-five participants were randomized into 25 blocks, with each block containing 3 participants. The first dose of study intervention was demonstrated by study staff and administered by mixing the product in approximately 125 mL of cool or room temperature water and having the participant drink it immediately before the investigational product settled. Participants were instructed to consume the investigational product in the morning. Bowel habit/daily diaries, stool collection supplies, and a 31-day supply of the study intervention were provided to the study participants during the baseline visit.

At Visit 3 (Week 1), previous bowel habit diaries, unused study interventions, and empty product packaging were collected, and compliance was calculated. New bowel habit diaries and stool collection supplies were provided to the participants. At Visit 4 (Week 4, the final study visit), previous bowel habit diaries, unused study products, and empty packaging were collected, and compliance was calculated. Participants collected fecal samples within 72 h prior to Visit 3 (Week 1) and Visit 4 (Week 4) and transferred them to the clinic site within 24 h of each collection.

Participants returned all sachets, including any open or unopened sachets, during both the 3rd and 4th visits. Compliance was calculated based on the amount of study product consumed compared to the total amount of study product expected to have been consumed for the given duration. Compliance for this study was considered acceptable if participants consumed an average of ≥80% of the study product for the given duration. Safety profiles were based on the safety analysis set (SAF; *n* = 75), while the full analysis set (FAS; *n* = 72) included participants who received at least one dose of the study product and had at least one outcome assessment after dosing and the per-protocol population (PP; *n* = 70) included only those who completed the study with overall compliance with study parameters.

### 2.3. Safety

The safety population (SAF) consisted of all participants who received at least 1 dose of the study product. Treatment-emergent adverse events (TEAEs) were defined as adverse events (AEs) with the onset time on or after the first dose of the study product. The number and percentage of participants and TEAEs are summarized based on Medical Dictionary for Regulatory Activities (MedDRA) System Organ Class (SOC), Preferred Term (PT), severity, and relationship to the study product by study arm. The relationship between the study intervention and each AE was assessed by the study clinician using their best judgment by examining and evaluating the participant based on the temporal relationship to the AE.

### 2.4. Microbiome Analysis

16Sv4 amplicons generated from fecal samples collected in OMNIgene-Gut kits (DNA Genotek) were sequenced on a MiSeq platform (Illumina, San Diego, CA, USA) at Microbiome Insights (Vancouver, BC, Canada). MiSeq-generated Fastq files were quality-filtered and clustered into 97% similarity operational taxonomic units (OTUs) using the mothur software package and the Greengenes v13.8 database [35]. The resulting dataset had 59,086 OTUs (including those occurring once with a count of 1 or singletons). An average of 30,860 quality-filtered reads were generated per sample. Sequencing quality for read one (R1) and read 2 (R2) was determined using FastQC 0.11.5. *Bifidobacterium* and *Akkermansia* were identified *a priori* as candidate genera that would increase in response to RPS consumption [29,30,36,37].

### 2.5. Self-Reported Bristol Stool Chart Scoring

Participants scored their bowel movements using the Bristol Stool Form Scale, also known as the Bristol Stool Chart (BSC), where Type 1 = constipation with hard, round stools, Type 2 = lumpy and sausage-like stools, Types 3 and 4 are soft, easily passed ‘normal’ stools, Type 5 = soft blobs of stool with clear-cut edges, Type 6 = stools with mushy consistency and ragged edges, and Type 7 = watery diarrhea [38]. In addition to quantifying extreme scores (i.e., Type 1 or Type 7), scores were grouped into constipation- (i.e., Type 1 or 2) or diarrhea-related (i.e., Type 6 or 7; Types 5, 6 or 7) bowel movement groups. Effects on either end of the scale can be muted when BSC scores are averaged. For example, a participant with equal numbers of Type 1 (constipation) and Type 7 (diarrhea) bowel movements would produce an average score of 4, falsely indicating that this participant experienced normal bowel movements. For this reason, BSC Type scores were not averaged and were kept discrete to evaluate the effects on both ends of the scale. The number of bowel movements meeting the categories above was quantified during the last seven recorded bowel movements to align with the microbiome analysis at 4 weeks, which was based on a stool sample collected 72 h before Visit 4.

### 2.6. Statistical Analysis

Change in relative abundance was determined by subtracting the baseline relative abundance from the relative abundance at week 4 for both *Bifidobacterium* and *Akkermansia*. Outliers were identified using the inter-quartile range (IQR) method, whereby changes in relative abundance greater and less than 1.5 times the IQR are considered outliers ([39,40,41,42]; Excel, Redmond, WA, USA). After removing outliers, RPS-dependent increases in bacterium levels were discretely compared to those in the placebo via Student’s *t*-tests (Excel). Bristol Stool Chart scores of bowel movements at 4 weeks were compared discretely between the placebo group and 3.5 g or 7 g treatment arms using 2-tailed Fisher’s exact tests (QuickCalcs, GraphPad Software, San Diego, CA, USA). In all instances, *p* < 0.05 was considered statistically significant.

## 3. Results

### 3.1. Participant Characteristics

One participant per treatment arm discontinued the study before Visit 4, and 2 participants in RPS high-dose arm were excluded due to non-compliance (<80% product consumption) and use of study-prohibited medication (Figure 1). Participants enrolled in the study were excluded if they had various gastrointestinal complaints, including a diagnosis of irritable bowel syndrome (IBS). Despite being healthy and excluded for these conditions, participants in each arm experienced a variety of bowel movement types during the run-in period (Table 3).

### 3.2. RPS Supplementation Increases Relative Abundance of Bifidobacterium

**Figure 2:**
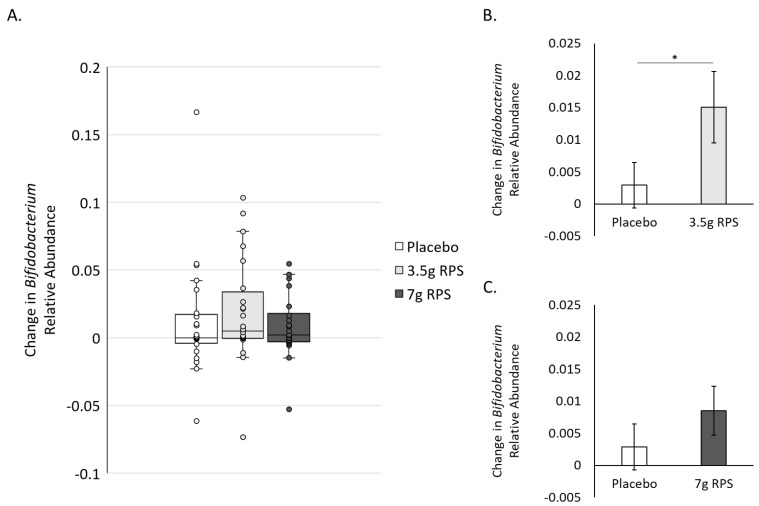
Increases in the relative abundance of *Bifidobacterium* after 4 weeks in response to placebo, 3.5 g RPS, or 7 g RPS. (**A**) Box and whisker plots revealing interquartile range (IQR; box), median (line inside box), and 1.5 times the IQR (error bars), as well as individual data points (circles). (**B**) The increase in the relative abundance of *Bifidobacterium* was significantly higher in the 3.5 g RPS group compared to the placebo arm (*p* = 0.038). (**C**) The increase in the relative abundance of *Bifidobacterium* was not significantly higher in the 7 g RPS group (*p* = 0.14) compared to the placebo arm (Student’s *t*-test, * *p* < 0.05).

*Bifidobacterium* is known to be the primary degrader of RPS [43], and the relative abundance of *Bifidobacterium* increased in stool samples from participants consuming 30 g of RPS daily for three months [30]. We, therefore, asked whether supplementation of 3.5 g of RPS for four weeks increased *Bifidobacterium* levels greater than placebo. Bifidogenic responses to 3.5 g RPS and placebo were variable (Figure 2A), and we identified three outliers in the 3.5 g RPS group and 4 outliers in the placebo group [39]. After removing outliers [40,41,42], we compared the mean change in relative abundance in *Bifidobacterium* in the 3.5 g RPS group to the placebo and found that the RPS increase in the relative abundance of *Bifidobacterium* was significantly greater than placebo (*p* = 0.038; Figure 2B). The relative abundance of *Bifidobacterium* also varied in the 7 g RPS group (Figure 2A), with two outliers identified. Mean comparison of changes in the relative abundance of *Bifidobacterium* in response to 7 g RPS did not show a statistically significant increase compared to placebo (*p* = 0.14; Figure 2C), although there was a trend towards increasing *Bifidobacterium* in the 7 g RPS group.

### 3.3. RPS Supplementation Increases Relative Abundance of Akkermansia

**Figure 3:**
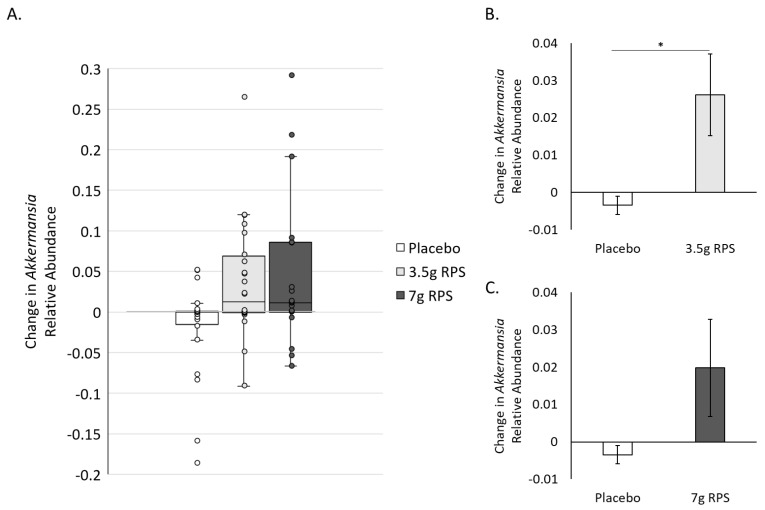
Increases in the relative abundance of *Akkermansia* after 4 weeks in response to placebo, 3.5 g RPS, or 7 g RPS. (**A**) Box and whisker plots revealing interquartile range (IQR; box), median (line inside box), and 1.5 times the IQR (error bars), as well as individual data points (circles). (**B**) The increase in the relative abundance of *Akkermansia* was significantly higher in the 3.5 g RPS group compared to the placebo arm (*p* = 0.014). (**C**) The increase in the relative abundance of *Akkermansia* was not significantly higher in the 7 g RPS group (*p* = 0.056) compared to the placebo arm (Student’s *t*-test, * *p* < 0.05).

Probiotic supplementation with strains of *Bifidobacterium* [44,45,46,47,48], supplementation with high amylose maize starch [49], and a blend of RS [37] has been shown to increase levels of *Akkermansia*, a genus associated with numerous health benefits [50,51]. We asked whether RPS might simultaneously increase the relative abundance of *Akkermansia*, potentially as a function of Bifidobacterium increases. Changes in the relative abundance of *Akkermansia* were variable (Figure 3A), yielding 1 outlier in the 3.5 g RPS group and 6 outliers in the placebo group [39]. After removing outliers [40,41,42], we compared the mean change in *Akkermansia* in the 3.5 g RPS group to the placebo and found that the RPS increase in the relative abundance of *Akkermansia* was significant (*p* = 0.014; Figure 3B). Relative abundance of *Akkermansia* also varied in the 7 g RPS group (Figure 3A), with 2 outliers identified. The mean comparison of changes in *Akkermansia* in response to 7 g RPS did not show a statistically significant increase compared to placebo (*p* = 0.056; Figure 3C), although there was a trend towards increasing *Akkermansia* in the 7 g RPS group.

### 3.4. RPS Supplementation Improves Constipation- and Diarrhea-Associated Bowel Movements

The frequency of different Bristol Stool Chart (BSC) types during the last seven bowel movements, corresponding to the week 4 *Bifidobacterium* and *Akkermansia* analysis time point, in the 3.5 g RPS and placebo arms were analyzed. There was a significant difference between the number of Type 7 (liquid consistency with no solid pieces) bowel movements at 4 weeks between the 3.5 g RPS and placebo arms (Table 4). Notably, there were no reports of Type 7 bowel movements in the 3.5 g RPS group. There were no significant differences in the number of other diarrhea-associated bowel movement scores, including Type 6 or 7 or Types 5, 6 or 7 (Table 4). While there was no difference in the number of Type 1 scores (separate hard lumps), there was a significant difference in the number of Type 1 or 2 (separate hard lumps and/or lumpy and sausage-like) scores at 4 weeks between the 3.5 g RPS and placebo arms (Table 4). We similarly compared BSC scores at 4 weeks between the 7 g RPS and placebo arms and found that only bowel movements of Type 5, 6 or 7 (soft blobs with clear-cut edges and/or mushy consistency with ragged edges and/or liquid consistency with no solid pieces) were significantly different (Table 5).

### 3.5. Safety

Overall, there were similar incidences of treatment-emergent adverse events (TEAEs) in treatment and placebo arms. Thirty-three participants experienced 56 TEAEs during the study period (Table 6). Most AEs were gastrointestinal (GI) in nature, with 16 participants reporting 24 (42.9%) GI-related AEs, followed by infections, including cold and flu, upper respiratory tract infections and urinary tract infections, reported by 12 participants reporting 12 (21.4%) of these AEs. All TEAEs assessed by the PI as being possibly related to the study products were reported within the gastrointestinal disorders SOC (11 participants [14.7%] reporting 17 events). Of the 11 participants having AEs possibly related to the study product, two participants received a placebo, two received a low dose, and seven received a high dose. The two participants receiving a placebo reported increased flatulence (*n* = 2), increased bloating (*n* = 1) and nausea (*n* = 1). In the low-dose arm, flatulence (*n* = 1) and bloating (*n* = 1) were reported as possibly related AEs. In the high-dose arm, the possibly related AEs were increased belching (*n* = 2), increased flatulence (*n* = 6), increased bloating (*n* = 2), and abdominal pain (*n* = 1). The gastrointestinal events reported as possibly related were similar in type between all groups, and the number of participants reporting these events was similar between the placebo arm and the low-dose arm. There were no serious AEs or deaths during the study period.

Treatment-emergent adverse events (TEAEs) in the full analysis set (FAS) population were neither serious nor fatal, and no TEAE led to study discontinuation. Participant numbers in the Severity and Relationship columns exceed the number in the Overall column because some participants experienced multiple events with discrete severity and/or relationship to the interventions. The majority of TEAEs were determined to be unrelated to the interventions, while less than a third were possibly related to the interventions, and no TEAEs were definitively related to the interventions.

## 4. Discussion

We tested the prebiotic properties of resistant potato starch (RPS) by administering 3.5 g or 7 g of RPS per day for 4 weeks and measuring the relative abundance of Bifidobacterium and scores of bowel movement consistency. Participants in the 3.5 g RPS arm demonstrated significantly higher increases in the relative abundance of Bifidobacterium compared to the placebo arm, along with fewer Type 7 (liquid consistency with no solid pieces) and Type 1 or 2 (separate hard lumps and/or lumpy and sausage-like) bowel movements compared to the placebo groups, thereby meeting prebiotic criteria [34]. Additionally, participants in the 3.5 g RPS arm displayed statistically significant increases in *Akkermansia* levels compared to the placebo group, further supporting the role that RPS can play in promoting the growth of health-associated microbes. These microbiome improvements are consistent with previous studies of RPS at high doses (i.e., 30 g/day), which increased *Bifidobacterium* [29,30,36] and promoted normal glucose metabolism [14]. Other sources of RS2, including high amylose maize starch [18,22] and green banana starch [52], have similarly been evaluated at high doses but studies evaluating low (i.e., <10 g/day) doses have not been reported.

Participants in the 7 g RPS arm had responses similar to those in the 3.5 g arm, but only improvements in diarrhea-associated bowel movement scores met statistical significance. Interestingly, while the relative abundance of *Bifidobacterium* increased in response to 7 g of RPS, the average increase was approximately 33% lower than what was observed in the 3.5 g arm. Curiously, this was also the case for *Akkermansia*. Several factors likely influenced this discrepancy, including the diets of participants, which were not controlled as part of the clinical trial. Dietary patterns improved predictions of gut microbiota species abundance in people undergoing daily monitoring of both diet and gut microbiome composition [53], suggesting that some of the variability in changes in *Bifidobacterium* and *Akkermansia* relative abundance could be due to participants consuming atypical foods. The median age of participants in the 7 g arm was 27.5 years, while the median ages in the placebo and 3.5 g arms were 37.5 and 41.5 years, respectively. Despite the mean ages being comparable, it is possible that age-related differences contribute to the lack of significant microbiome and bowel movement improvements in this group.

The genus *Akkermansia*, including *Akkermansia muciniphilia*, primarily utilizes host mucus-derived glycans as an energy source [54], making our findings that low-dose RPS administration increased *Akkermansia* levels surprising. *Akkermansia muciniphilia* has gained attention for promoting healthy metabolism and has been developed into probiotic and postbiotic formats [50,51]. Clinical trials evaluating the effects of administering probiotic strains of *Bifidobacterium* also show increases in *Akkermansia* levels [44,45,46,47,48], suggesting that the RPS-dependent increases could be a consequence of *Bifidobacterium* activities or other RPS-dependent changes in the microbial environment. In the absence of *Bifidobacterium* increases, high doses of prebiotic inulin produced modest increases in *Akkermansia* in hemodialysis patients [55], as did high doses of high amylose maize starch in insulin-resistant individuals [49] and an RS blend containing RPS, green banana starch, and apple pectin [37], indicating that prebiotics may support *Akkermansia* independent of probiotic-associated bacterium increases. Intriguingly, the magnitude of the increase in the relative abundance of *Akkermansia* (0.026 +/− 0.011) was greater than the *Bifidobacterium* increase (0.015 +/− 0.006) for the 3.5 g RPS dose (Figure 2A and Figure 3A). Cross-feeding between *Bifidobacterium* and butyrate-producing bacteria has been hypothesized to enhance mucin secretion, thereby increasing *Akkermansia*, suggesting that the beneficial actions of *Bifidobacterium* are more important in promoting *Akkermansia* levels than relative *Bifidobacterium* abundance per se [46].

This novel study is the first to demonstrate increases in health-associated bacteria *Bifidobacterium* and *Akkermansia* in response to a low dose of RS, as were the improvements in stool form. Previous studies using RS from a variety of sources have found improvements in bowel movement characteristics at higher dosage levels (i.e., 17–30 g/day; [10,11,12,13]). Oligosaccharide prebiotics like lactulose reduce constipation by increasing stool bulk via microbial cell growth and increasing luminal osmotic pressure in the small intestines, which can lead to diarrhea when administered at too high a dose (i.e., 20–40 g/day) or for too long a duration [56]. Participants consuming a 3.5 g RPS dose had significantly fewer diarrhea- and constipation-associated bowel movements after 4 weeks compared to the placebo group (Table 5), indicating that supplementation with RPS relieves constipation without promoting excessively soft stools. Importantly, Type 7 (liquid diarrhea with no solid pieces) bowel movements were completely absent in the 3.5 g arm after 4 weeks, demonstrating that this dose of RPS effectively normalizes stool form. Reductions in both diarrhea- and constipation-associated bowel movements are uncommon among dietary fiber intervention studies, and poorly fermentable psyllium is the best example of a fiber that promotes improvements in participants who experience both overly firm and overly loose stools, effects attributed to the water-holding and fecal bulking properties of this fiber [57]. To our knowledge, this is the first report of a low-dose fermentable fiber normalizing stool form in a clinical study of healthy individuals.

There are limitations to this study, including the short duration. Bowel movement records were not kept beyond the 4-week timepoint, making it difficult to connect changes in bowel movement scores to changes in the composition of the gut microbiota derived from a stool sample collected at the end of the study. We compensated for this limitation by analyzing the final seven bowel movements recorded for each participant, which we reasoned was an appropriate approximation of the bowel movements consistent with the microbiota composition at 4 weeks. A longer study would also be useful to confirm the stability of these findings. It is possible that baseline levels of bacteria and stool consistency reflect cessation of vitamin and/or dietary supplement consumption, and future studies would benefit from recruiting participants earlier for a longer ‘wash-out’ period. The short duration also limited the investigation into the effects of RPS on the gut microbiota, which has pronounced beneficial effects at higher doses for longer durations [30]. Another limitation was the exclusion of individuals clinically diagnosed with digestive symptoms, which was necessary to evaluate the efficacy of formulation into dietary supplements. Our study indicates that RPS could be beneficial for individuals suffering from IBS, especially those who alternate between diarrheal and constipated bowel movements or experience a ‘mix’ of symptoms [58]. Future studies could benefit from a cross-over design, in which participants consume each dose with wash-out periods in between, facilitating dose-response comparisons within individuals. Such a study could better account for the influences of dietary preferences, host glycan production, and baseline microbiome-dependent effects.

## 5. Conclusions

We demonstrate that RPS has a prebiotic effect at a 3.5 g daily dose, which is comparable to prebiotic oligosaccharide doses (i.e., 3–7 g/day; [59]). RPS significantly increased *Bifidobacterium* and *Akkermansia* levels and decreased diarrhea- and constipation-associated bowel movements compared to the placebo, meeting the International Scientific Association for Probiotics and Prebiotics (ISAPP) consensus criteria for the definition of a prebiotic [34]. *Bifidobacterium* and *Akkermansia* increases in the 7 g RPS arm trended positively towards statistical significance compared to the placebo, consistent with the 3.5 g dose, and improvements in bowel movement scores were significantly different from the placebo group in both treatment arms. RPS was safe and well tolerated at both a 3.5 g daily dose and a 7 g daily dose. Future studies examining the other host benefits at low RPS doses and benefits in healthy people and patients with IBS are warranted.

## Figures and Tables

**Table 1:** Baseline demographic data for the FAS population.

	7 g RPS Dose	3.5 g RPS Dose	Placebo	Total
Number of Participants	24	24	24	72
Age (Year) ^#^				
Mean (SD)	36.6 (15.85)	38.5 (14.63)	39.1 (16.50)	38.1 (15.49)
Median	27.5	41.5	37.5	35.0
Min, Max	19, 64	20, 66	19, 66	19, 66
Sex				
Male	9 (37.5%)	6 (25.0%)	7 (29.2%)	22 (30.6%)
Female	15 (62.5%)	18 (75.0%)	17 (70.8%)	50 (69.4%)
Race				
Indigenous	0 (0.0%)	0 (0.0%)	1 (4.2%)	1 (1.4%)
Asian	3 (12.5%)	3 (12.5%)	0 (0.0%)	6 (8.3%)
Black or African Canadian	1 (4.2%)	0 (0.0%)	0 (0.0%)	1 (1.4%)
White	19 (79.2%)	21 (87.5%)	23 (95.8%)	63 (87.5%)
Other	1 (4.2%)	0 (0.0%)	0 (0.0%)	1 (1.4%)
Ethnicity				
Hispanic or Latino	0 (0.0%)	0 (0.0%)	2 (8.3%)	2 (2.8%)
Not Hispanic or Latino	24 (100%)	24 (100%)	22 (91.7%)	70 (97.2%)
Height (cm)				
Mean (SD)	168.73 (8.919)	165.60 (7.351)	167.87 (9.682)	167.40 (8.684)
Median	167.05	165.10	166.25	166.20
Min, Max	151.5, 186.9	153.5, 186.5	151.3, 184.5	151.3, 186.9
Weight (kg)				
Mean (SD)	69.12 (13.836)	73.40 (12.281)	71.20 (13.811)	71.24 (13.257)
Median	65.20	74.60	71.00	71.40
Min, Max	47.4, 102.8	54.1, 98.2	47.0, 105.8	47.0, 105.8
BMI (kg/m^2^)				
Mean (SD)	24.10 (3.420)	26.78 (4.291)	25.14 (3.735)	25.34 (3.938)
Median	23.25	26.90	24.30	24.80
Min, Max	18.8, 31.8	19.6, 33.8	19.8, 33.3	18.8, 33.8

Age, sex, race, ethnicity, height, weight, and body mass index (BMI) for full analysis set (FAS) participants at baseline. ^#^ Age calculated based on the date of informed consent signed and date of birth. Standard deviation (SD) is indicated.

**Table 2:** Study visits and timeline of events.

	Name	Events	Day
Visit 1	Screening	Inclusion/exclusion criteria reviewed, informed consent obtained, medical history and exam, vital signs and blood safety sample taken, fecal collection kits and diaries provided	−30 to −14 days
Visit 2	Baseline	Inclusion/exclusion criteria, dietary supplements and medications, and medical history reviewed, pregnancy test (females only), fecal sample and diaries collected, randomization	Day 0
Visit 3	Week 1	Fecal sample and diaries collected, unused study products collected, compliance calculated, and product re-dispensed, review medication and dietary supplement history, new fecal collection kits and diaries dispensed	~Day 8 (+2 day window)
Visit 4	Week 4	Fecal sample and diaries collected, unused study products collected and compliance calculated, review medication and dietary supplement history	~Day 29 (+3 day window)

Visit number, corresponding event, and respective day number for the major activities during the trial.

**Table 3:** Baseline Bristol Stool Chart (BSC) scores.

	Type 1	Type 2	Type 3	Type 4	Type 5	Type 6	Type 7	Total
Placebo	12	34	50	190	92	32	15	429
3.5 g RPS	9	27	100	209	53	46	11	455
7 g RPS	34	49	50	136	109	40	5	423

Baseline (BSC) scores for all bowel movements recorded during the run-in period (15–17 days, depending on the participant).

**Table 4:** The presence of bowel movements scored according to the Bristol Stool Chart (BSC) at 4 weeks in the 3.5 g RPS and placebo arms.

	Type 1	Not Type 1	*p* Value
Placebo	3	165	0.99
3.5 g RPS	2	166	
	Type 1 or 2	Not Type 1 or 2	
Placebo	23	145	0.0085
3.5 g RPS	9	159	
	Type 7	Not Type 7	
Placebo	6	162	0.03
3.5 g RPS	0	168	
	Type 6 or 7	Not Type 6 or 7	
Placebo	13	155	0.35
3.5 g RPS	19	149	
	Type 5, 6 or 7	Not Type 5, 6 or 7	
Placebo	49	119	0.26
3.5 g RPS	39	129	

The last seven bowel movements recorded for each participant (corresponding with a 4-week timepoint) were scored according to the BSC and compared between the 3.5 g RPS and Placebo treatment groups using Fisher’s exact test.

**Table 5:** Presence of bowel movements scored according to the Bristol Stool Chart (BSC) at 4 weeks in the 7 g RPS and placebo arms.

	Type 1	Not Type 1	*p* Value
Placebo	3	165	0.13
7 g RPS	8	146	
	Type 1 or 2	Not Type 1 or 2	
Placebo	23	145	0.44
7 g RPS	26	128	
	Type 7	Not Type 7	
Placebo	6	162	0.51
7 g RPS	3	151	
	Type 6 or 7	Not Type 6 or 7	
Placebo	13	155	0.26
7 g RPS	7	147	
	Type 5, 6 or 7	Not Type 5, 6 or 7	
Placebo	49	119	0.026
7 g RPS	28	126	

The last seven bowel movements recorded for each participant (corresponding with a 4-week timepoint) were scored according to the BSC and compared between the 7 g RPS and Placebo treatment groups using Fisher’s exact test.

**Table 6:** Summary of TEAEs in the FAS population.

	7 g RPS Dose		3.5 g RPS Dose		Placebo		Total	
	Participants (*n* = 25)	Events (*n* = 14)	Participants (*n* = 25)	Events (*n* = 22)	Participants (*n* = 25)	Events (*n* = 20)	Participants (*n* = 72)	Events (*n* = 56)
Overall	10 (40.0%)	14 (100%)	11 (44.0%)	22 (100%)	12 (50.0%)	20 (100%)	33 (44.0%)	56 (100%)
Serious	0	0	0	0	0	0	0	0
Fatal	0	0	0	0	0	0	0	0
Discontinuation	0	0	0	0	0	0	0	0
Severity								
Mild	9 (36.0%)	12 (85.7%)	6 (24.0%)	9 (40.9%)	10 (40.0%)	15 (75.0%)	25 (33.3%)	36 (64.3%)
Moderate	1 (4.0%)	1 (7.1%)	8 (32.0%)	12 (54.5%)	4 (16.0%)	5 (25.0%)	13 (17.3%)	18 (32.1%)
Severe	1 (4.0%)	1 (7.1%)	1 (4.0%)	1 (4.5%)	0	0	2 (2.7%)	2 (3.6%)
Relationship								
Related	0	0	0	0	0	0	0	0
Possibly related	2 (8.0%)	2 (14.3%)	7 (28.0%)	10 (45.5%)	2 (8.0%)	5 (25.0%)	11 (14.7%)	17 (30.4%)
Not related	9 (36.0%)	12 (85.7%)	7 (28.0%)	12 (54.5%)	12 (48.0%)	15 (75.0%)	28 (37.3%)	39 (69.6%)

## Data Availability

The data that support the findings of this study are available from MSP Starch Products Inc., but restrictions apply to the availability of these data, which were used under license for the current study, and so are not publicly available. Data are, however, available from the authors upon reasonable request and with permission of MSP Starch Products Inc. Individual patient data is unavailable as per the informed consent form and the interventional study protocol registration.
